# Involvement of Intestinal Epithelium Aryl Hydrocarbon Receptor Expression and 3, 3′-Diindolylmethane in Colonic Tertiary Lymphoid Tissue Formation

**DOI:** 10.3390/ijms251810153

**Published:** 2024-09-21

**Authors:** Erika L. Garcia-Villatoro, Zachary S. Bomstein, Kimberly F. Allred, Evelyn S. Callaway, Stephen Safe, Robert S. Chapkin, Arul Jayaraman, Clinton D. Allred

**Affiliations:** 1 Department of Nutrition, Texas A&M University, College Station, TX 77843, USA; 2 Department of Nutrition, University of North Carolina Greensboro, Greensboro, NC 27412, USA; 3 Department of Veterinary Physiology and Pharmacology, Texas A&M University, College Station, TX 77840, USA; 4 Program in Integrative Nutrition & Complex Diseases, Texas A&M University, College Station, TX 77843, USA; 5 Department of Chemical Engineering, Texas A&M University, College Station, TX 77843-3127, USA

**Keywords:** colon, tertiary lymphoid tissues, aryl hydrocarbon receptor, DIM, intestinal epithelium

## Abstract

Tertiary lymphoid tissues (TLTs) are adaptive immune structures that develop during chronic inflammation and may worsen or lessen disease outcomes in a context-specific manner. Immune cell activity governing TLT formation in the intestines is dependent on immune cell aryl hydrocarbon receptor (AhR) activation. Homeostatic immune cell activity in the intestines is further dependent on ligand activation of AhR in intestinal epithelial cells (IECs), yet whether AhR activation and signaling in IECs influences the formation of TLTs in the presence of dietary AhR ligands is not known. To this end, we used IEC-specific AhR deletion coupled with a mouse model of dextran sodium sulfate (DSS)-induced colitis to understand how dietary AhR ligand 3, 3′-diindolylmethane (DIM) influenced TLT formation. DIM consumption increased the size of TLTs and decreased T-cell aggregation to TLT sites in an IEC-specific manner. In DSS-exposed female mice, DIM consumption increased the expression of genes implicated in TLT formation (Interleukin-22, *Il-22*; CXC motif chemokine ligand 13, *CXCL13*) in an IEC AhR-specific manner. Conversely, in female mice without DSS exposure, DIM significantly reduced the expression of *Il-22* or *CXCL13* in iAhRKO mice, but this effect was not observed in WT animals. Our findings suggest that DIM affects the immunological landscape of TLT formation during DSS-induced colitis in a manner contingent on AhR expression in IECs and biological sex. Further investigations into specific immune cell activity, IEC-specific AhR signaling pathways, and dietary AhR ligand-mediated effects on TLT formation are warranted.

## 1. Introduction

The small and large intestines are persistently exposed to a diverse array of microorganisms, dietary metabolites, or otherwise bioactive exogenous compounds through the ingestion and digestion of foods and the excretion of feces [1]. Consequently, both organs are equipped with a vast network of lymphoid tissue equipped to cope with and adapt to immunogenic threats, and due to the extent and quantity of such tissues throughout the GI tract, the intestines are considered the largest immune organ in the body [2]. The development of lymphoid tissue such as Peyer’s patches and isolated lymphoid follicles (ILFs) throughout the intestines occurs during embryogenesis and the post-natal period, and due to their encapsulated and highly organized germinal centers, such structures are considered secondary lymphoid organs (SLOs) [2,3]. In contrast to the programmed nature of SLOs, aggregates of follicular dendritic cells (FDCs), B cells, and T cells known as tertiary lymphoid tissues (TLTs) have been shown to develop across the intestinal epithelium at sites of chronic or severe inflammation [3,4].

Though dependent on the disease and tissue of origin, TLT lymphogenesis may be generalized as follows (i) priming of stromal cells by inflammatory cytokines inducing a fibroblast-like phenotype, (ii) release of chemokines and adhesion factors from primed stroma, resulting in attraction and adherence of lymphocytes and dendritic cells, and (iii) “maturation” of lymphoid-activated stromal sites, whereby high endothelial venules (HEVs) allow continual clustering of lymphocytes and eventual FDC accumulation at the TLT site [4,5]. Continual clustering and maturation of immune cell aggregates at TLT sites in the absence of an encapsulated structure allows direct exposure of maturing and formed TLTs to antigens, providing a more efficient immune response than that of SLOs, though such a response may be beneficial or detrimental in a disease-specific context [4,6]. In colorectal cancer (CRC), for example, tumors exhibiting a high degree of microsatellite instability (MSI) may develop intra and peritumoral TLTs due to mutation-induced inflammation. Preclinical models of induced CRC demonstrate that TLT formation may augment intestine-specific antitumoral immune responses by enhancing tumor-infiltrating CD3+ lymphocyte recruitment to the tumor site [7,8]. Indeed, TLT formation during CRC is associated with a better prognosis and a lower likelihood of tumor recurrence in clinical cases [9,10,11]. Furthermore, in multiple preclinical models where inflammation is induced, TLT formation is protective against colitis. Contrarily, TLT formation during Crohn’s disease (CD) correlates with disease severity and may worsen disease-originating autoimmunity [5,12,13]. Collectively, this underscores the complexity between the formation and adaptive role of TLTs in various inflammatory contexts [14].

Both nutritive and non-nutritive dietary constituents exert anti-inflammatory and immunoregulatory effects on the intestines and by extension, contribute towards protection against diseases with inflammatory etiologies [15,16,17,18]. A growing body of evidence demonstrates that diet and microbially derived metabolites contribute towards anti-inflammatory effects through ligand binding by the aryl hydrocarbon receptor (AhR) [19,20,21,22,23]. The AhR, expressed widely throughout the GI tract, is a promiscuous nuclear receptor that upon binding a ligand, translocates to the nucleus, where it exerts genomic effects [21]. AhR has long been considered a detoxification receptor due to its ability to initiate the transcription of Phase I metabolism enzymes following ligand binding, such as cytochrome p450 superfamily enzymes Cyp1a1 and Cyp1b1 [24]. Today, the AhR’s role in physiology extends beyond that of oxidative metabolism of ligands and is understood to be a critical component of mucosal immunity [25]. Specifically, TCRγδ intra-epithelial lymphocytes (IELs) require AhR activation for the maintenance of their immunoregulatory functions in the small and large intestines. Due to their indispensable role in perforin and granzyme production and subsequent protection from bacterial insults, pathogenic microorganism colonization in the intestinal lumen ensues in AhR^−/−^ mice [26,27]. Steady-state AhR activity is also required for the maintenance of Il-22 production by RORγt^+^ ILCs, with the RORγt transcription factor recruitment to the nucleus facilitated by the activation of AhR [28]. Steady-state Il-22 production in this regard is a critical requirement for promoting IEC-originating Antimicrobial Peptide (AMP) production and enhancing goblet cell mucus production [29,30]. To this end, murine models of experimentally induced colitis demonstrate that the provision of dietary AhR ligands restores homeostatic Il-22 and ILC3-mediated immune cell signaling, resulting in a less severe colitis phenotype [28]. Activation of AhR by exogenous ligands in intestinal epithelial cells (IECs) is equally important in intestinal health [29]. In intestinal epithelial stem cells (IESCs), AhR regulates IESC proliferation and differentiation by inhibiting Wnt–B-catenin signaling [31]. Activation of AhR in intestinal epithelia has been further shown to maintain tight junctions and promote their formation during models of chemical insult [32,33,34]. IEC metabolism of AhR ligands directly governs their availability to submucosal immune cells that require AhR activation for immunogenic functions [28,35]. For example, constitutive cyp1a1 expression in intestinal epithelia of mice resulted in extensive clearance of AhR ligands, resulting in a significant reduction in colonic ILC3s and Il-22 production, mimicking a quasi AhR-deficient state [28].

Inflammation-dependent development of TLTs involves AhR-driven processes, such as RORγt transcriptional activity on group 3 innate lymphoid cells (ILC3s), Il-22 production, and T-helper 17 (Th17) cell activity [36]. 3, 3′-diindolylmethane (DIM) is a byproduct of gastric digestion of indole-3-carbinol (I3C), a phytochemical widely found in cruciferous vegetables like broccoli and kale and a widely documented AhR ligand [37]. Provision of DIM to the diet has been shown to ameliorate DSS-induced colitis through AhR-dependent immunoregulatory and lymphogenic pathways, as well as through downregulation of inflammatory cytokines implicated in TLT formation [38,39]. Yet, whether the provision of dietary DIM specifically affects TLT formation or the specific immune response elicited by the structures during intestinal inflammation has not been resolved. Further, whether dietary AhR ligands influence the initiation of TLT formation in an IEC-specific manner is not known, which is important due to the role of IEC-specific AhR activity in governing downstream immune cell activity. Thus, the objective of our study was to use mice with IEC-specific AhR deletion to understand how the provision of DIM in the diet would affect TLT formation during a model of DSS-induced colitis through AhR-mediated signaling processes at the intestinal epithelium. We hypothesized that DIM consumption would reduce the severity of DSS-induced colitis and alter associated TLT formation, effects that would be lost in mice with IEC-specific AhR deletion.

## 2. Results

### 2.1. DIM Lessened the Severity of DSS-Induced Colitis without Altering Intestinal Permeability

**Figure 1:**
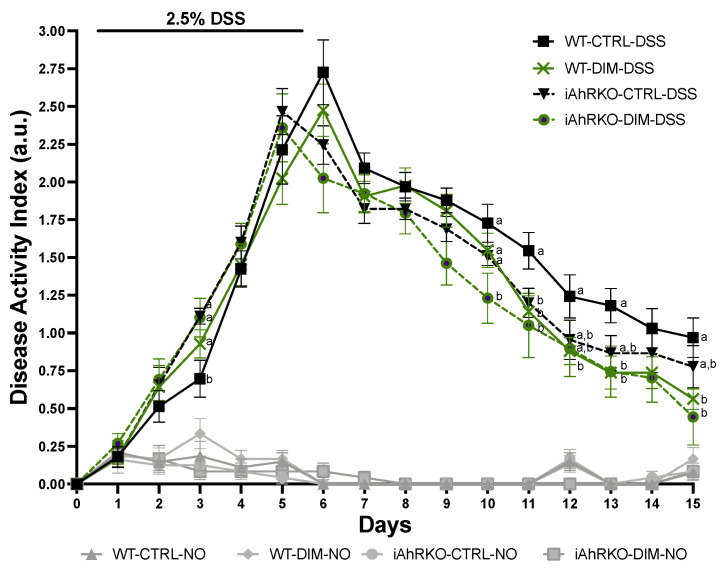
Effect of the loss of AhR in IECs on clinical signs of acute colitis. Disease Activity Index (DAI), a composite measure of weight loss, stool consistency, and presence of blood in stool. Data presented indicate the mean ± SEM. For each indicated day, groups without a common letter differ; *p* < 0.05.

**Figure 2:**
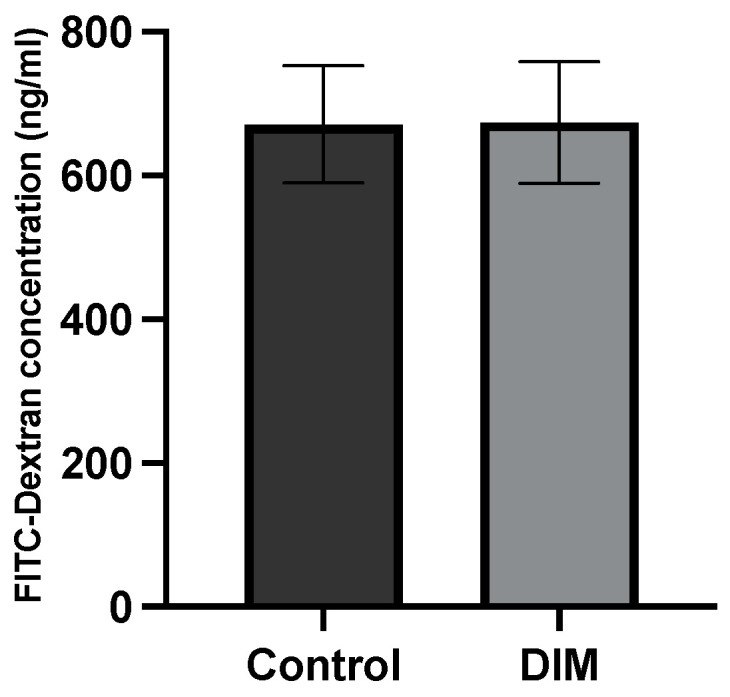
Serum concentration of FITC-dextran at termination. Effect diet (DIM supplementation) compared to control diet. Data presented indicate the mean ± SEM.

Five-day administration of 2.5% dextran sulfate sodium (DSS) induced acute colitis demonstrated by diarrhea, visible (macroscopic) blood in feces, colon shortening, and decreased body weight (Figure 1). Mice were terminated ten days after the fifth day of DSS. DAI was measured from baseline (day 0) to termination (day 15). At the end of this experiment, WT animals fed a DIM-supplemented diet showed a significant (*p* < 0.05) reduction in DAI compared to WT mice fed a semi-purified (control) diet (Figure 1). DAI scores of iAhRKO-CTRL-DSS mice were not significantly different than any other group at the end of the 15-day measurement period. Measurement of intestinal permeability after FITC-dextran administration demonstrated that the presence of DIM in the diet did not have a significant effect on gut permeability, irrespective of sex or genotype (Figure 2).

### 2.2. DIM Consumption Affects the Quantity, Size, and T-Cell Density of TLTs 

**Figure 3:**
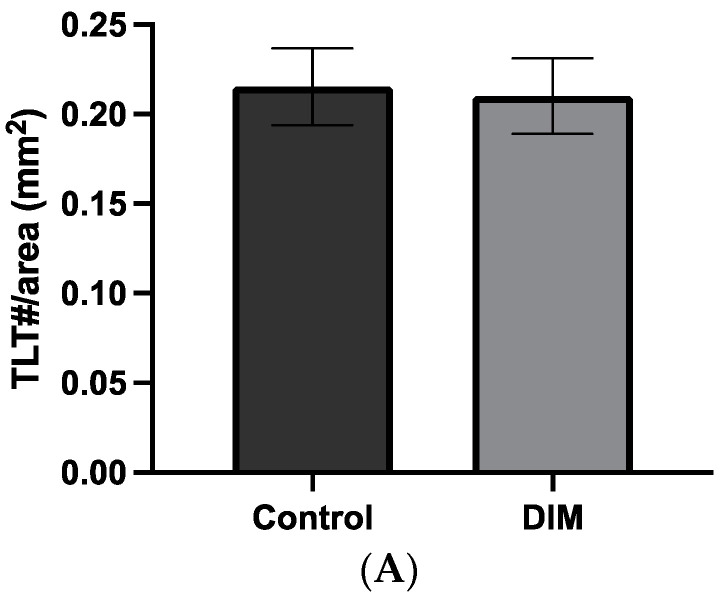

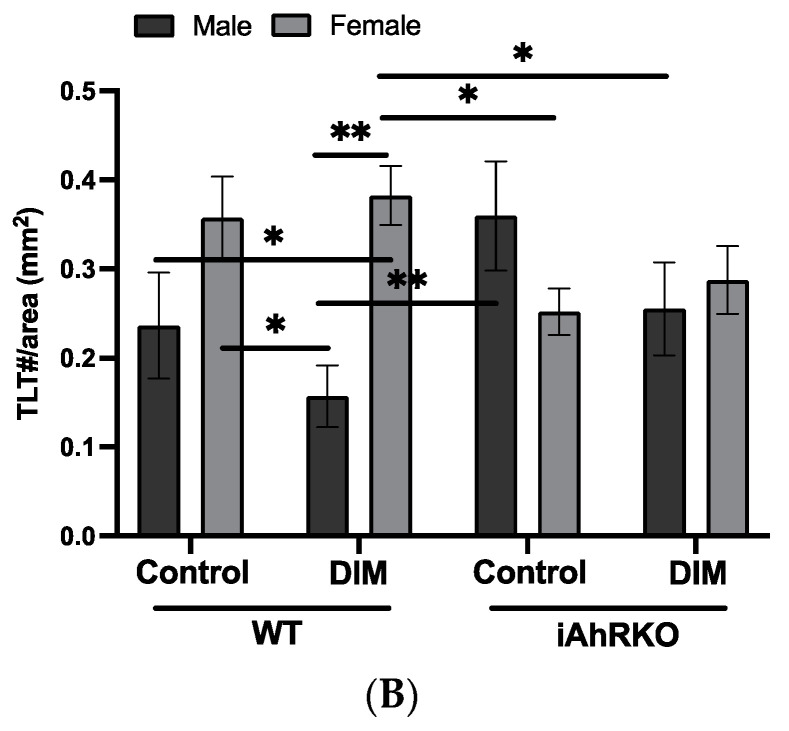
Number of tertiary lymphoid tissues (TLT/measurable area (mm^2^). (**A**) Effect of diet (DIM supplementation) compared to control diet. (**B**) Effect of genotype, diet, and sex in mice treated with DSS. Data presented indicate the mean ± SEM. * *p* < 0.05, ** *p* < 0.01.

When considering all mice in the study, no significant changes in the quantity of de novo tertiary lymphoid tissue (TLT #/area) between mice receiving a DIM-supplemented diet vs. control were observed (Figure 3A). In DSS-treated animals, a three-way ANOVA analysis (*p* = 0.0183) revealed a significant interaction between sex and genotype (*p* = 0.0021) and between sex and diet (*p* = 0.0633) (Figure 3B). In females, the formation of TLTs was not affected by the presence of DIM in the diet of WT or iAhRKO groups when analyzed separately. In males, DIM non-significantly reduced the formation of TLTs in both WT and iAhRKO mice when compared to their corresponding control diet-fed counterparts. When the significant interaction between sex, genotype, and diet was considered, the formation of TLT significantly increased in WT females fed a DIM-supplemented diet compared to WT males (Figure 3B). Further, WT males fed DIM had significantly fewer TLTs than their iAhRKO counterparts fed a control diet, while females lacking AhR (IEC) expression fed a control diet (KC) had significantly fewer TLTs than WT females fed DIM (Figure 3B).

**Figure 4:**
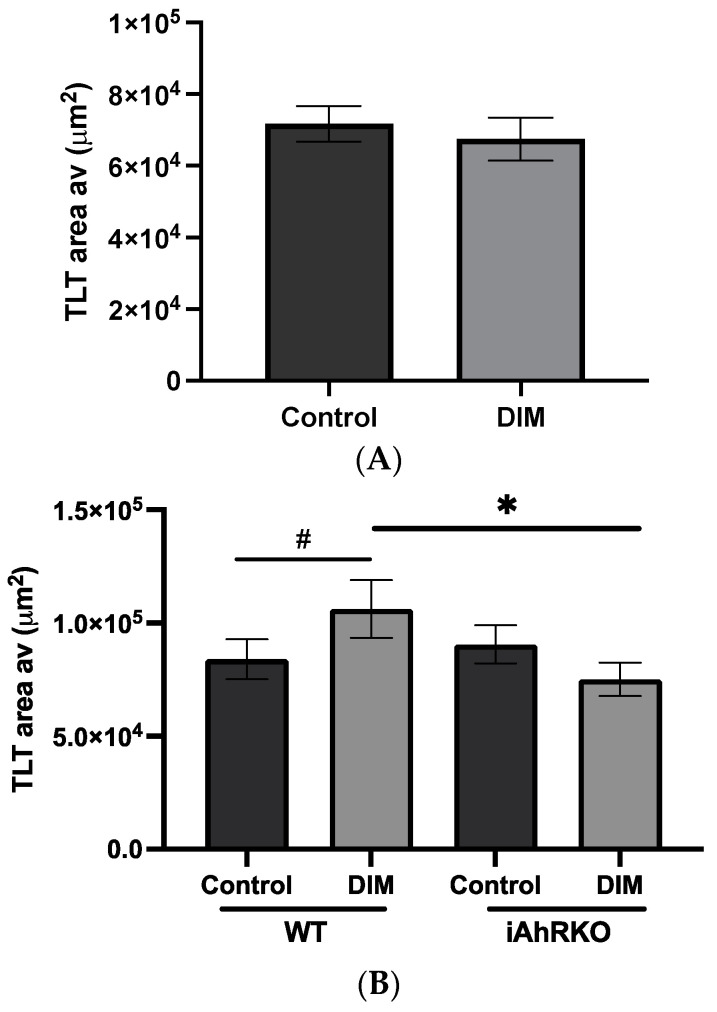
Size of Tertiary Lymphoid tissues (average area of TLT (µm^2^). (**A**) Effect of diet (DIM supplementation) compared to control diet. (**B**) Effect of genotype and diet (DIM vs. control) in mice treated with DSS. Data presented indicate the mean ± SEM. # *p* < 0.075, * *p* < 0.05.

**Figure 5:**
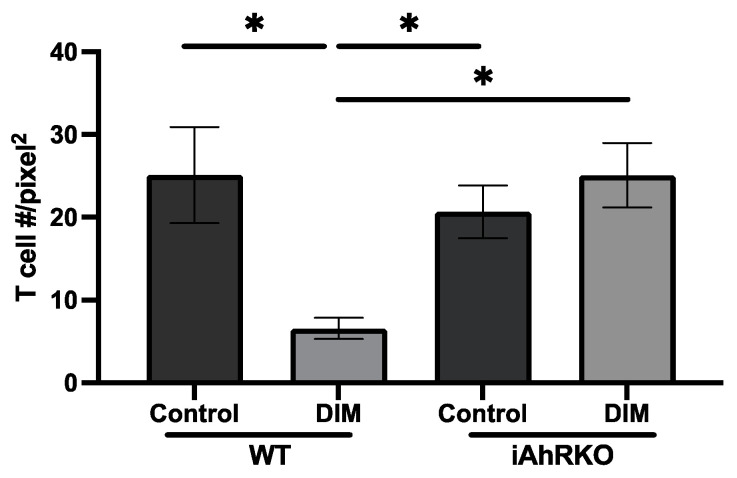
Effect of genotype, sex, and diet in immune cell density within TLT in DSS-treated animals. Effect of genotype and diet in T-cell density within TLT (Genotype*Diet, ANOVA (KW) *p* = 0.0112. Data presented indicate the mean density (Cell #/pixel^2^) ± SEM; * *p* < 0.05.

When comparing all mice consuming DIM to those that did not, no significant differences in TLT size were observed (Figure 4A). However, in WT mice, DIM increased the size of TLTs (*p* = 0.0767) compared to the control diet, while in iAhRKO mice, the size of TLTs was not affected by DIM (Figure 4B). Within identified TLTs, we further assessed the comparative density of T cells between groups of DSS-treated animals. As illustrated in Figure 5, the DIM-supplemented diet significantly reduced the T-cell density compared to the control diet in WT mice (*p* = 0.0397). This effect was lost in iAhRKO mice fed a DIM diet.

### 2.3. Inflammation and Barrier Function Gene Expression in Isolated Colon Crypts (ICCs)

**Figure 6:**
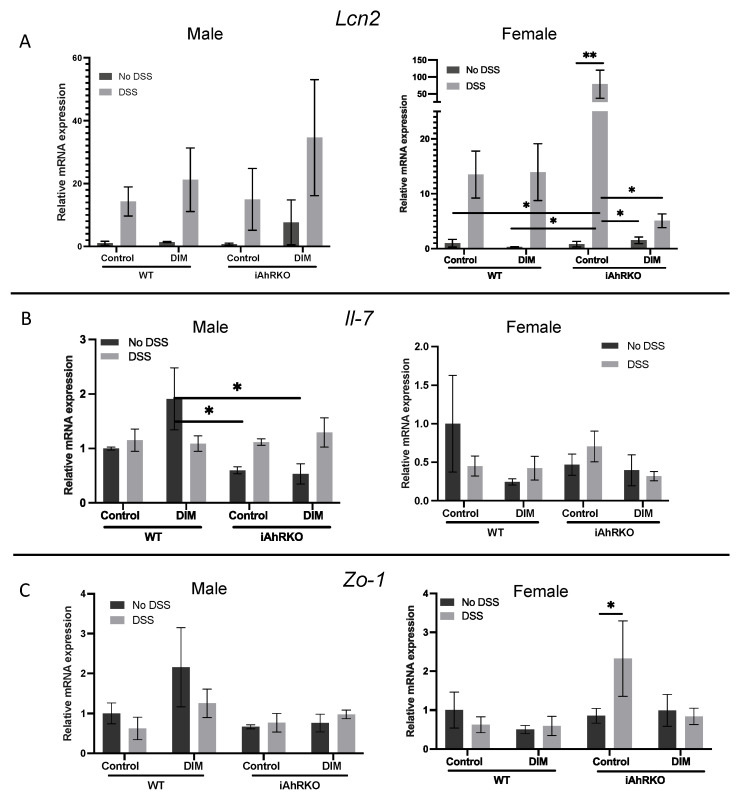

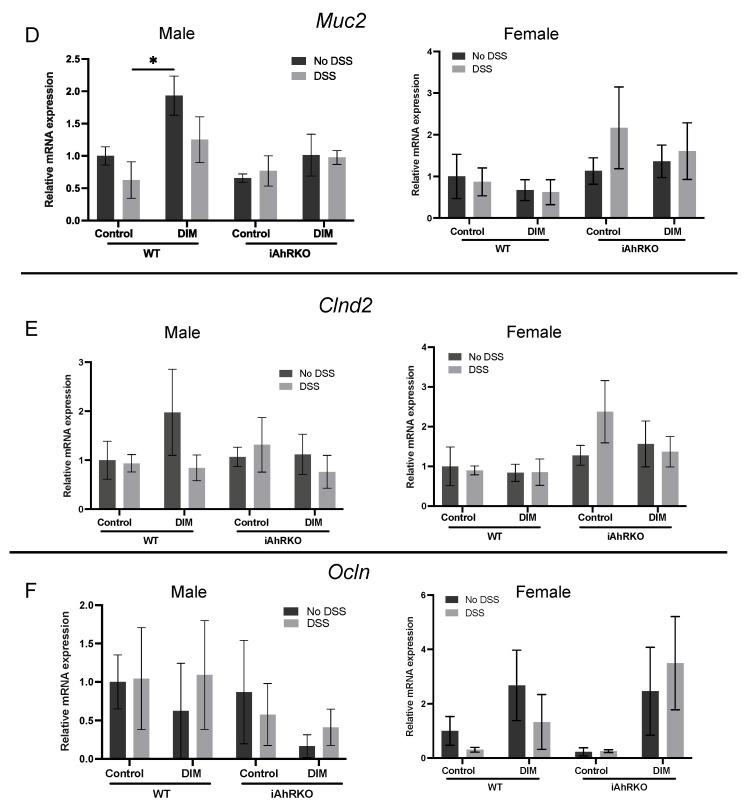
Effect of diet, genotype, DSS, and sex in the expression of IEC-derived genes from isolated colonic crypts (ICCs). (**A**) Relative expression of *Lcn2* (Lipocalin-2), 2-way ANOVA; male (DSS effect, *p* = 0.0215) and female (DSS effect, *p* = 0.0224). (**B**) Relative expression of *Il-7* (IL-7), 2-way ANOVA; male (Interaction between diet/genotype and DSS, *p* = 0.0197). (**C**) Relative expression of *Zo-1* (ZO-1/Tjp1). (**D**) Relative expression of *Muc2* (Muc2). (**E**) Relative expression of *Cldn2* (Claudin-2). (**F**) Relative expression of *Ocln* (Occludin). Data are expressed as fold change relative to WT control, n = 3–4, * *p* < 0.05, ** *p* < 0.01.

To understand the contribution of IECs separately from other subtypes of cells in the colonic mucosa to intestinal barrier function and host response to inflammation after DIM exposure, we analyzed RNA from IECs and measured the expression of genes involved in inflammation and intestinal barrier function. *Lcn2* (Neutrophil Gelatinase-associated Lipocalin) is a gene expressed during colonic inflammation and whose translated product (Lipocalin-2) is used as a clinical indicator of ulcerative colitis [40,41]. In female iAhRKO mice only, DIM consumption combined with DSS exposure significantly decreased IEC expression of *Lcn2* relative to female iAhRKO mice exposed to DSS without DIM (Figure 6A). *Il-7* is an inflammatory cytokine that is upregulated during DSS-induced colitis [42]. Surprisingly, DSS did not significantly affect the expression levels of *Il-7* regardless of diet, genotype, or sex compared to vehicle controls (Figure 6B). In mice without DSS exposure, DIM increased the expression of *Il-7* in WT males compared to their control-fed counterparts (interaction between diet, genotype, and DSS, *p* = 0.0197) (Figure 6B). No significant effects were observed in females when comparing experimental diets and treatments in both genotypes (Figure 6B).

*Zo-1*, *Cldn2*, *Ocln*, and *Muc2* code for proteins requisite for the synthesis and maintenance of intestinal epithelial tight junctions and mucus secretion. In male mice, *Zo-1* expression was not affected by diet, genotype, or exposure to DSS. Expression of *Zo-1* was significantly upregulated (*p* = 0.0393) in iAhRKO female mice that received DSS compared to non-DSS in females (Figure 6C). WT males fed DIM had an increase (+90%) in *Muc2* expression at a basal state compared to mice fed a control diet, while expression levels of *Muc2* were not affected by the genotype, nor exposure to DSS, in females (Figure 6D). *Cldn2* and *Ocln* expression did not significantly differ by diet, genotype, or DSS exposure (Figure 6E,F). 

### 2.4. Mucosal Expression of Genes Involved in TLT Initiation 

**Figure 7:**
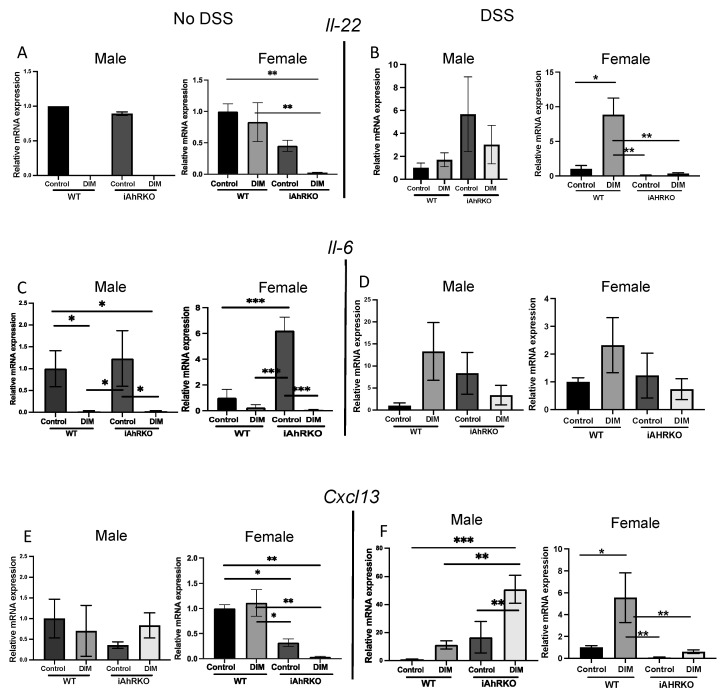
Effect of genotype, diet, DSS, and sex in the expression of Il-22, Il-6, and CXCL13 from MS. (**A**) Relative expression of Il-22 (IL-22)/No DSS. Male (N/A, 0 = undetectable) and female (*p* = 0.0096, one-way ANOVA). (**B**) Relative expression of Il-22 (IL-22)/DSS. Male (*p* = 0.3469, one-way ANOVA) and female (*p* = 0.0021, KW). (**C**) Relative expression of Il-6 (IL-6)/No DSS. Male (*p* = 0.0021, KW) and female (*p* = 0.0004, one-way ANOVA). (**D**) Relative expression of Il-6 (IL-6)/DSS. Male (*p* = 0.2915, one-way ANOVA) and female (*p* = 0.1426, KW). (**E**) Relative expression of Cxcl13 (Cxcl13)/No DSS. Male (*p* = 0.5244, one-way ANOVA) and female (*p* = 0.0051, one-way ANOVA). (**F**) Relative expression of Cxcl13 (Cxcl13)/DSS. Male (*p* = 0.0015, one-way ANOVA) and female (*p* = 0.0106, one-way ANOVA). Data are expressed as fold change relative to WT control, n = 3–4, * *p* < 0.05, ** *p* < 0.01 and *** *p* < 0.001.

We then sought to measure the expression of mucosal genes involved in the initiation of TLT lymphogenesis. IL-22 activity during chronic inflammation potentiates TLT formation through mediating stromal cell chemokine secretion and subsequent lymphocyte aggregation [6,41]. *Il-22* expression in female iAhRKO mice consuming a DIM-supplemented diet was significantly decreased relative to WT female mice consuming either a control or a DIM-supplemented diet (Figure 7A). During DSS exposure, DIM consumption led to significantly greater *Il-22* expression relative to WT females consuming a control diet, while such a difference was not evident in female iAhRKO counterparts (Figure 7B). Further, expression of *Il-22* was significantly upregulated in WT females fed the DIM-supplemented diet compared to the control diet (790%, *p* = 0.0005) (Figure 7B).

IL-6 is an inflammatory cytokine integral to the intestinal innate immune response to inflammation [42,43]. As shown in Figure 7C, male mice that received DIM in the diet had lower expression levels of *Il-6* in non-DSS-exposed mice regardless of the genotype. Also, in non-DSS mice, mucosal *Il-6* expression in iAhRKO animals consuming the control diet was significantly greater than both WT groups, as well as in female iAhRKO mice consuming DIM (Figure 7C). Significantly, although *Il-6* expression was affected by DIM consumption in the absence of DSS, neither diet nor genotype significantly impacted *Il-6* levels in male and female mice treated with DSS (Figure 7D).

As one of many chemokines secreted by stromal cells during cytokine stimulation, CXCL13 is particularly important in B-cell attraction during TLT initiation. In males, *CXCL13* expression was unaffected by diet or genotype in non-DSS-exposed mice. However, in females, loss of AhR in IECs significantly reduced *CXCL13* expression by 70% (*p* = 0.0426) in control-fed mice and by 97% (*p* = 0.0063) in DIM-fed mice compared with WT-control fed females (Figure 7E). Compared to control-fed WT males that received DSS, loss of AhR in IECs significantly increased *CXCL13* expression by more than 10-fold when DIM was present in the diet (*p* = 0.0193) and by 16- and 50-fold (*p* = 0.0002) when compared to iAhRKO control and DIM-fed animals, respectively (Figure 7F). In WT females, DIM increased *CXCL13* expression compared with control-fed mice by 6-fold (*p* = 0.0090) (Figure 7F). Likewise, *CXCL13* expression was significantly downregulated (−90%, *p* = 0.0090) in iAhRKO/ control-fed mice and by 40% in iAhRKO/DIM-fed mice compared with their WT-control fed counterparts (Figure 7F).

## 3. Discussion

TLTs develop at sites of chronic, disease-induced inflammatory niches and have context-specific effects on gastrointestinal inflammation [4]. Using an inducible, intestinal epithelial cell-specific AhR knockout mouse model (iAhRKO), we demonstrated that the provision of a diet rich in AhR ligand DIM during DSS-induced colitis reduced colitis severity. We further showed that DIM administration increased TLT size while decreasing T-cell density in an IEC AhR-specific manner. These findings were accompanied by distinct transcriptional profiles in IECs linking cytokine, chemokine, and inflammatory markers to the findings in a sex, genotype, and diet-specific manner. 

DAI was measured daily during DSS exposure to evaluate colitis severity. In all groups, DSS significantly increased DAI scores relative to mice who received no DSS, irrespective of sex, diet, or genotype. Compared with WT animals that received a ligand-depleted diet (control), DIM supplementation reduced DAI in both WT and knockout mice. Administration of DIM (1–20 mg/kg) before DSS or AOM/DSS exposure ameliorated clinical and histological characteristics of DSS-induced colitis in a dose-dependent manner [38,39]. While our study resulted in some similar observations using a dietarily relevant dose of DIM (20 mg/kg BW), we demonstrate that many of the effects of DIM are not dependent on AhR activity in IECs. Interestingly, mice with IEC-specific AhR deletion had an earlier maximum DAI compared to WT mice. Existing literature suggests that IEC-specific AhR-KO worsens DSS-induced colitis [43]. While the earlier maximum DAI seen in iAhRKO could suggest that IEC-specific AhR deletion causes a more rapid disease induction relative to WT mice, it is hard to conclude without sufficient analyses of markers of tissue inflammation at various time points. This will be a focus of future studies.

With a background of DSS exposure, WT mice consuming DIM exhibited significantly increased TLT size, relative to WT mice consuming the control diet, and this effect was lost in iAhRKO mice. This suggests that DIM-mediated changes, in this regard, were reliant on the IEC expression of AhR. TLT size is positively correlated with the extent and severity of local inflammation [44,45]. While this may suggest DIM enhanced local inflammation in the presence of induced colitis, we further found that DIM consumption amongst WT mice strongly reduced T-cell density at TLT sites, an effect that similarly disappeared in iAhRKO mice. These effects may be explained by the fact that in addition to its role in ILC3 and IEL activity, AhR activation is crucial to the proliferation and activity of certain FDCs and B-cell subsets [46,47,48]. Thus, provision of ample DIM in our model may have accented T-cell-dependent and -independent B-cell proliferation in response to DSS-induced colitis. Further, DIM has been reported to ameliorate the severity of DSS-induced colitis through AhR-dependent regulation of the T-helper 17 (Th17)/Foxp3+ Regulatory T-cell (Tregs) axis, favoring the suppression of pro-inflammatory Th17 activity [38,39]. Thus, DIM may reduce Th17/B-cell interactions at TLT sites while supporting an augmented adaptive B-cell response to DSS exposure, explaining the greater TLT area observed with a marked reduction in T-cell density. As these effects disappeared in iAhRKO mice, this suggests that IEC-specific AhR activation by DIM was necessary to produce such effects, a finding that warrants further investigation. 

The initiation of TLTs during intestinal inflammation is associated with distinct cytokine and chemokine signatures, one of which is the secretion of the chemokine CXCL13 by intestinal stromal cells [3,4,6]. Our findings demonstrate that during DSS exposure, female mice consuming a DIM diet exhibit marked upregulation of the *CXCL13* gene in the intestinal mucosa, an effect that disappears in female mice with IEC-specific AhR deletion. Interestingly, in mice without DSS exposure, *CXCL13* was reduced in iAhRKO females, irrespective of diet suggesting that steady-state *CXCL13* expression in IECs is an AhR-dependent process. In mice, IEC-specific overexpression of CXCL13 mediated B-cell aggregation and lymphoid structure formation, suggesting that regulation of CXCL13 in the intestinal epithelium may be critical to early-stage lymphocyte aggregation during TLT lymphogenesis [49]. Though our findings suggest that DIM-dependent *CXCL13* expression is an AhR-dependent process, only one study to date has reported on its transcriptional regulation through the nuclear receptor. AhR ligand Benzo[a]pyrene has been shown to upregulate CXCL13 in A549 lung epithelial cells in vitro [50]. As we further showed that *CXCL13* in female iAhRKO mice is significantly downregulated irrespective of diet in non-DSS animals, our findings novelly point to AhR-mediated regulation of *CXCL13* expression in female mice and by extension TLT lymphogenesis. Further, recently published work from our lab has shown that TLT area and T-cell density following DSS exposure, CXCL13-dependent events, differ in a sex-dependent manner [51]. Therefore, further investigation into the AhR-dependent regulation of CXCL13 in IECs is warranted. 

Like CXCL13, we found that during DSS exposure, female mice consuming a DIM diet exhibit marked upregulation of the *Il-22* gene expression in intestinal mucosa, an effect that disappears in female mice with IEC-specific AhR deletion. In a mouse model of viral laryngeal TLT induction, IL-22−/− mice failed to develop TLTs, an effect that was experimentally determined to be related to Il-22-mediated promotion of stromal cell CXCL13 secretion [14]. This supports our findings and suggests the importance of the IL-22/CXCLl3 axis in TLT formation in our model [49]. AhR regulatory processes govern intestinal-wide Il-22 activity through RORγt-dependent ILC3 activation, as well as through regulatory effects on IEC-mediated intestinal barrier function and anti-microbial peptide production [28,29,30]. Interestingly, in a model of DSS-induced colitis where I3C was administered in the absence of IEC AhR expression, Il-22-dependent immune cell function, and subsequent anti-inflammatory activity could not be rescued, pointing to the importance of AhR expression in IECs to multicompartmental regulation of Il-22-mediated immune system activity [52]. Our findings are in support of this notion, as IEC-specific AhR deletion inhibited *Il-22* upregulation observed following DIM consumption in WT mice. 

Il-6 secretion by IECs is important in innate immune system response to intestinal epithelium insult, particularly in models of DSS-induced colitis [53,54]. While we observed no differences in IEC *Il-6* expression in mice exposed to DSS, we observed distinct differences in steady-state *IL-6* expression within males and females in our study. Specifically, DIM consumption in male mice significantly reduced *IL-6* expression relative to control, irrespective of IEC AhR expression. To our knowledge, this is the first time demonstrating such findings in healthy mice, though several models of DIM exposure combined with DSS-induced colitis show that administration of the compound reduces inflammation with accompanying decreases in Il-6 production [38] and ([39] p. 3). Due to the role of the cytokine in innate immune responses, our finding may suggest that dietary DIM protects the intestinal epithelium from inflammatory insults in an AhR-dependent fashion [55]. In females, IEC-specific AhR deletion resulted in a significant increase in steady-state *lL-6* production, an effect that was rescued by DIM consumption. Sexual dimorphism exists with respect to host immunity, with females possessing higher immune cells constituting the body’s innate response to insult [56,57,58]. As innate immune cell populations such as ILC3s are dependent on AhR signaling, dysregulated or underactive immune responses may worsen inflammation in a non-diseased state [25]. If true, this would further explain our finding that DIM consumption inhibited IEC *IL-6* expression, even in iAhRKO females. Taken together, regulation of AhR at the intestinal epithelium may play important, sex-specific roles in steady-state IL-6 production.

*Lcn2* (Neutrophil Gelatinase-associated Lipocalin) is a gene expressed during colonic inflammation and whose translated product (Lipocalin-2) is used as a clinical indicator of ulcerative colitis [40,41]. Expectedly, DSS exposure increased *Lcn2* expression relative to mice not exposed to DSS. Interestingly, we demonstrated that expression of *Lcn2* was significantly reduced in female iAhRKO mice receiving DIM relative to iAhRKO control-fed female mice, while no such difference was observed in males. This finding suggests that in female mice specifically, DIM may attenuate inflammation through a mechanism dependent on IEC AhR activity. As these effects were not observed in WT mice, the precise role of Lcn2 during acute inflammation in the context of AhR activation in IECs remains to be elucidated.

Inflammatory stimuli drive intestinal TLT formation and as DSS-induced colitis is a function of intestinal barrier disruption, we measured the expression of genes involved in intestinal barrier formation and maintenance [5,55]. Interestingly, we found no significant differences in the expression of *Cldn2* or *Ocln*. Meanwhile, male WT mice consuming DIM exhibited significantly greater *Muc2* expression compared to male WT mice exposed to DSS but consuming a control diet. Further, female iAhRKO mice consuming a CD exposed to DSS exhibited higher *Zo-1* expression relative to their non-DSS-exposed counterparts. Our results may be explained by the timing of TLT analysis. We allowed a recovery period following stopping DSS exposure, which allows TLTs time to form. However, this, time between that last dose of DSS and the measurement of IEC gene expression may have been too long to effectively capture changes in barrier function gene expression. This is further supported by the fact that though we showed DIM alleviated the severity of colitis (measured via DAI), we did not detect any significant differences in intestinal permeability measured via a FITC-dextran assay. 

In conclusion, our findings demonstrate that DIM consumption reduces the severity of DSS-induced colitis and affects the formation, size, and T-cell density of TLTs in a manner dependent on the function of AhR in the intestinal epithelium. Furthermore, we demonstrated that the expression of genes in IECs crucial to initiating TLTs are enhanced by DIM consumption in an IEC- and sex-dependent fashion. Subsequent investigations are warranted to clarify the specific adaptive immune response evoked by dietary AhR ligands in the intestinal microenvironment to understand how such a response may benefit or worsen colitis-associated inflammatory outcomes. 

## 4. Materials and Methods

### 4.1. Animal Model

**Figure 8:**
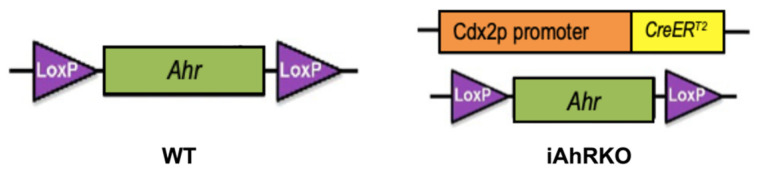
Mouse model used CDX2P^CreT2^ x AhR^f/f^.

**Figure 9:**
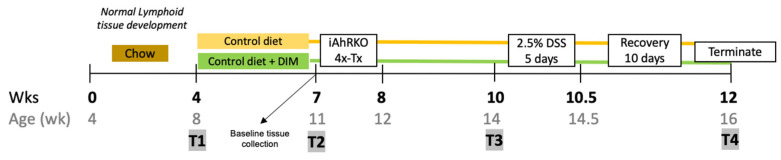
Study design.

An intestinal epithelial cell-specific AhR knockout mouse model (CDX2P-Cre-ER^T2^ x AhR^f/f^, iAhRKO) (IMSR Cat# JAX:022390, RRID:IMSR_JAX:022390) with AhR^f/f^ littermates (IMSR Cat# JAX:006203, RRID:IMSR_JAX:006203) as WT controls was used for this study [59,60] (Figure 8). Both male and female mice were enrolled after weaning (4 weeks of age, n = 104) (Figure 9). Mice were fed a standard rodent diet (Harlan Teklad Cat#8604) for four weeks until sexual maturity (8 weeks) was reached (Figure 9). Mice were then divided into two experimental diet groups (Control and DIM). The control diet was a semi-purified powder rodent diet containing 10% fat (Research Diets Inc., New Brunswick, NJ, USA. AIN-76A, Cat# D100001). The experimental diet consisted of the same control diet supplemented with 3, 3’-Diindolylmethane (DIM) at 20 mg/kg BW (Sigma-Aldrich, St. Louis, MO, USA, Cat# D9568). DIM (63.75 mg DIM/kg Diet, %w/w) was mixed into the control diet by the investigator as described in previously published methods [61]. Mice received food and water ad libitum; food was replaced every 48 h throughout the study. Mice were housed on a 12 h/12 h light–dark cycle with five mice per cage. Bodyweight was assessed weekly. Experimental diets were provided at the start of the fourth week and continued for three weeks before the induction of AhR knockout. A subset of animals was terminated at week 7 to establish a baseline number of TLTs while the remaining animals received a daily intraperitoneal injection of tamoxifen for four days. Dextran sodium sulfate (DSS) was administered 15 days after tamoxifen injections to ensure effective AhR knockout by a complete crypt turnover [62,63]. 

### 4.2. Induction of Colitis 

At week 10, 2.5% dextran sodium sulfate (DSS: MP Biomedicals, Santa Anna, CA, USA, Cat# 9011-18-1, MW 36–50 kDa) was provided ad libitum in sterile drinking water for 5 days to induce acute colitis [64,65]. Fresh DSS water was provided every 48 h. Animals in the control group received normal drinking water. Bodyweight, fecal consistency, and macroscopic fecal blood scores were recorded daily until the end of the study to assess the disease activity index (DAI). The DAI scoring system was adapted from previously published sources [66,67]. Briefly, body weight loss percentage was evaluated as 0 if weight gain of 0–1% loss occurred, 1 if 1–5% loss, 3 if 10–15% loss, and 4 if 15% or more loss occurred compared to baseline measurements at Day 0. Rectal bleeding (macroscopic fecal blood) was scored as 0 if a normal stool was present, 1 if the pellet was soft but formed, 2 if the pellet was very soft, 3 if diarrhea (no pellet) was observed, and 4 if dysenteric diarrhea (blood in diarrhea) was present. Rectal bleeding was scored as 0 no bleeding, 2 blood was visible in the stool (red/dark pellet), and 4 gross macroscopic bleeding from the anus. All procedures were performed under protocol number 2018-0205 approved by the Institutional Animal Care and Use Committee at Texas A&M University. 

### 4.3. Fecal and Tissue Collection

Fecal samples were collected at four different time points, as indicated in Figure 9 (T1–T4). Animals were single-housed for up to 2 h, and feces were collected and flash-frozen in liquid nitrogen and stored at −80 °C until analysis. In the 12th week of the study, food was withdrawn overnight, 8 h prior to termination, and FITC-dextran was given four hours prior to termination via oral gavage (more details below). Mice were anesthetized using ketamine/xylazine (100 mg/kg body mass, IP injection), and blood was collected by cardiac puncture. Blood was allowed to clot for 45 min, then serum was collected and stored at −80 °C. Colons were resected, flushed with PBS, the contents were collected, and length and weight were assessed. Cecal contents were collected and flash-frozen in liquid nitrogen. The colon was opened longitudinally with the mucosal side facing up along the mesenteric fat line. The distal colon was sectioned into two equal parts, placed on a cassette, and fixed in 10% neutral buffered formalin (Sigma-Aldrich, St. Louis, MO, USA, Cat# HT501128) for 3 h. Tissues were rinsed in cold PBS three times and stored in 70% ethanol at room temperature prior to processing and embedding. The proximal colon was divided into two equal parts to study the differential expression of genes produced by IECs and the rest of the intestinal wall. The mucosa was scraped, homogenized, and stored in RNA-lysis buffer at −80 °C, while the other section was processed to extract RNA from isolated colonic crypts as previously described by Davidson L. et al. [68]. The spleen and liver were removed, weighed, and fixed in 10% neutral buffered formalin for 24 h. 

### 4.4. Intestinal Permeability Assessment 

Intestinal permeability was measured with the fluorescent marker fluorescein isothiocyanate–dextran (FITC-dextran) (Sigma, St. Louis, MO, USA, Cat# FD4) dissolved in sterile PBS at a concentration of 80 mg/dL. 600 mg/kg BW of 80 mg/dL FITC-dextran solution was gavaged into each mouse four hours prior to termination. Blood collected was left to clot for 45 min, in the dark, and centrifuged at 12,000× *g* for 4 min. Serum FITC-dextran levels were determined using a Fluorometer with an excitation of 485 nm and an emission of 528 nm (20 nm bandwidth). FITC-dextran was serially diluted and used as a standard (0, 125, 250, 500, 1000, 2000, 4000, 8000 ng/mL). 

### 4.5. Histological Assessment of Tertiary Lymphoid Tissues 

Distal colons were processed, embedded, sectioned (4 mm), and H&E stained. With the mucosal side facing up, crypts were serially sectioned transversally to allow the visualization of lymphoid tissues. An Aperio ScanScope^TM^ slide scanner (Leica, Wetzlar, Germany) was used at 20× magnification to digitize slides. QuPath v. 0.1.2 was used to identify, quantify, and assess the area of tertiary lymphoid tissues (TLT) [69]. Raw data were collected and processed using Microsoft Excel^®^ (v. 16.37). The auto-detection (wand) tool in QuPath was used to measure the entire surface area (measurable area, MA) of the distal colon (µm^2^). TLTs in the distal colon mounts were visualized as dense and defined clusters of lymphoid tissues without a capsule of connective tissue [70]. After identification of TLTs, individual area measurements/TLT were recorded. For each slide, the total number of TLTs and the area (size) of each TLT were recorded. Mean values obtained were calculated and used for subsequent statistical analysis. 

### 4.6. Immuno-Histo-Fluorescence Staining of T Cells

Formalin-fixed, paraffin-embedded, 4 mm serial sections of distal colon from DSS-treated animals were deparaffinized, rehydrated, and after antigen retrieval in Citrate Buffer (10 mM, pH 6), sections were blocked for one hour at room temperature with 5% goat serum (Abcam, Cambridge, UK, Cat# ab7481, RRID: AB_2716553) and 5% donkey serum (Sigma-Aldrich, St. Louis, MO, USA, Cat# D9663, RRID: AB_2810235). Sections were incubated overnight at 4 °C with Armenian hamster (IgG) anti-CD3e monoclonal antibody (Thermo Fisher Scientific, Waltham, MA, USA, Cat# 14-0031-82, RRID: AB_467049, 1:50 dilution). Sections were washed and incubated with secondary antibodies for one hour at room temperature. Secondary antibodies used were goat anti-Armenian hamster (IgG) H&L-Alexa Fluor 647 (Abcam Cat# ab173004, RRID:AB_2732023, 1:500 dilution). Washes between stages (3x, 5 min each) were performed in PBSt (PBS 1X with 0.02% Tween 20). Primary antibodies were not used for negative control sections. After the final wash, slides were coverslipped using ProLong Gold Antifade Mountant with DAPI (Invitrogen, Waltham, MA, USA, Cat# P36930) and cured overnight until dry. T-cell-stained tissue images focused on the TLT area and were acquired at 20X magnification using the all-in-one fluorescent digital imaging microscope (BZ-X700, Keyence Corporation, Osaka, Japan) with the Cy5 channel (“red”) to capture the Alexa Fluor 647 dye signal. 

### 4.7. Gene Expression Analysis

Mucosal scrapings (MSs) and isolated colonic crypts (ICCs) were homogenized in RNA-lysis buffer and stored at −80 °C. Quick-RNA MiniPrep (Zymo Research, Irvine, CA, USA, Cat# R1054) was used to isolate total RNA according to the manufacturer’s instructions, including DNase treatment. RNA from ICCs and MSs was eluted with 40 mL and 25 mL of RNase-free water, respectively. The Transcriptor First Strand cDNA Synthesis Kit (Roche, Basel, Switzerland, Cat# 04896866001 was used to reverse-transcribe samples into cDNA according to the manufacturer’s instructions. LightCycler 480 SYBR Green I Master Mix (Roche, Cat# 04707516001) was used in quantitative real-time PCR experiments following manufacturer instructions in a LightCycler 480 using gene-specific primers to measure relevant functional IEC genes, such as tight junctions (TJs) and pro/anti-inflammatory cytokines (Supplemental Table S1). 

### 4.8. Statistical Analysis

Statistical analyses and figures were generated using GraphPad Prism version 8.1.2 for macOS, Graph Pad Software (La Jolla, CA, USA www.graphpad.com, accessed on 13 August 2024). Parametric or non-parametric methods were used to compare means according to compliance with normality (Shapiro–Wilk test). ROUT (Q = 1.0%) was used to exclude outliers. Parametric methods included an unpaired *t*-test for comparing two means or one-way ANOVA followed by Tukey’s multiple comparisons test for comparing three or more means. Two-way ANOVA was used to analyze interaction effects between variables. Non-parametric methods used included the Mann–Whitney U (MW) test for comparing two means or the Kruskal–Wallis (KW) test along with Dunn’s multiple comparisons test to compare three or more means. Values listed are group means, and error bars represent SEM. Differences among groups were considered statistically significant when the *p*-value was ≤0.05. Within figures, * indicates *p* ≤ 0.05, ** indicates *p* ≤ 0.01, *** indicates *p* ≤ 0.001, and the absence of * indicates *p*-values > 0.05.

## Data Availability

The data presented in this study are openly available in [FigShare] [https://figshare.com/articles/dataset/Data_for_DIM_Paper/26549821?file=48366358] (accessed on 13 August 2024).

## Supplementary Materials

The following supporting information can be downloaded at: https://doi.org/10.6084/m9.figshare.25395322 (accessed on 13 August 2024).
